# Electroacupuncture promotes synaptic plasticity in rats with chronic inflammatory pain–related depression by upregulating BDNF/TrkB/CREB signaling pathway

**DOI:** 10.1002/brb3.3310

**Published:** 2023-11-10

**Authors:** Pu Yang, Haiyan Chen, Tian Wang, Hong Su, Jing Li, Yujun He, Shengyong Su

**Affiliations:** ^1^ The First School of Clinical Medicine Guangxi University of Chinese Medicine Nanning Guangxi China; ^2^ Department of Nursing The First Affiliated Hospital of Guangxi University of Chinese Medicine Nanning Guangxi China; ^3^ Faculty of Acupuncture, Moxibustion and Tuina Guangxi University of Chinese Medicine Nanning Guangxi China; ^4^ Department of Acupuncture and Moxibustion The First Affiliated Hospital of Guangxi University of Chinese Medicine Nanning Guangxi China; ^5^ Guangxi Key Laboratory of Molecular Biology of Preventive Medicine of Traditional Chinese Medicine Nanning Guangxi China

**Keywords:** BDNF/TrKB/CREB‐signaling pathway, chronic inflammatory pain, depression, electroacupuncture, hippocampus, synaptic plasticity

## Abstract

**Background:**

Chronic inflammatory pain (CIP) frequently coincides with depression among patients. The onset and development of pain and depression are associated with altered neural synaptic plasticity. Electroacupuncture (EA) can effectively relieve CIP and depression. However, the underlying mechanisms have not been fully illustrated.

**Objective:**

To explore whether EA can relieve CIP and depression by regulating hippocampal synaptic plasticity, and the present study offers foundational evidence for the efficacy of EA in treating CIP‐related depression (CIPD).

**Methods:**

Rats were divided into four groups: 0.9% normal saline group, complete Freund's adjuvant (CFA) group, CFA + duloxetine group, and CFA + EA group. Pain hypersensitivity was detected by mechanical withdrawal threshold and thermal paw withdrawal latency, and the depression level was gauged using the open field test, the sucrose preference test, and the forced swimming test. The morphology of the hippocampal neurons was observed using Nissl staining. The protein expression levels of synuclein (Syn), postsynaptic density protein‐95 (PSD‐95), brain‐derived neurotrophic factors (BDNFs), tyrosine‐protein kinase B (TrKB), p‐TrkB, cAMP response element binding protein (CREB), and p‐CREB were measured by western blotting and immunofluorescence staining. BDNF and TrkB mRNA expression were detected using quantitative real‐time polymerase chain reaction (PCR) (qRT‐PCR). The content of 5‐hydroxytryptamine (5‐HT) and γ‐aminobutyric acid (GABA) was detected using enzyme‐linked immunosorbent assay, and the glutamic acid (Glu) content was determined using the ultraviolet colorimetry method. The hippocampal neuron ultrastructure was observed using transmission electron microscopy.

**Results:**

EA could alleviate CIP and related depressive behaviors as well as protect the hippocampal neuronal structure from damage and regulate 5‐HT/GABA/Glu levels in the hippocampus. Additionally, EA could significantly increase the expression of synapse‐associated proteins such as PSD‐95 and Syn by activating the BDNF/TrKB/CREB signaling pathway.

**Conclusion:**

EA improves pain and depressive behaviors in CIPD rats, and the mechanism may be related to synaptic plasticity mediated by the BDNF/TrKB/CREB signaling pathway.

## Graphical abstract

CFA injection can induce pain sensitivity and depression‐like behavior in adult male SD rats. Electroacupuncture has demonstrated antidepressant and analgesic properties, which can be attributed to its ability to enhance hippocampal synaptic plasticity by mitigating neuronal damage, modulating neurotransmitters, regulating synaptic proteins, and activating the BDNF/TrkB/CREB signaling pathway.

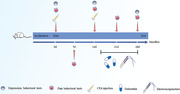

## 1 INTRODUCTION

Chronic pain, a prolonged condition lasting beyond 12 weeks, encompasses neuropathic and inflammatory pain; this multifaceted condition involves subjective sensory and emotional components (Mills et al., 2019; Pitzer et al., 2019). Depression frequently cooccurs with multiple clinical conditions, resulting in challenging disease prognoses (Read et al., 2017). Among individuals diagnosed with depression, the incidence of chronic pain ranges from 51.8% to 59.1% (Corruble & Guelfi, 2000), whereas the prevalence of depression in those with chronic pain can be as high as 85% (Doan et al., 2015). The coexistence of chronic pain and depression has been shown to mutually reinforce each other, resulting in disease exacerbations and challenges in treatment (Haleem, 2019). As a significant global burden of disease, the association between chronic inflammatory pain (CIP) and depression has garnered attention. CIP leads to the enhanced activation of neurobiological processes underlying depression, influenced by inflammatory responses (Guo et al., 2023; Pinho‐Ribeiro et al., 2017). Several studies have elucidated shared biological pathways and molecular mechanisms underlying both conditions, including neurotransmitters, neuroinflammatory factors, brain‐derived neurotrophic factors (BDNFs), central sensitization, glutamate, and its receptor subtypes (Lottering & Lin, 2021; Meda et al., 2022). Therefore, understanding the molecular mechanisms of CIP‐related depression (CIPD) offers potential targets and solutions for its diverse clinical manifestations.

Chronic pain is closely linked to maladaptive changes in nociceptive pathways, involving abnormal phenomena like ectopic action potentials and increased synaptic transmission. Furthermore, neuroplastic alterations, including synaptic connectivity loss, the formation of new synaptic circuits, and neuroimmune interactions, may also contribute to its development (Costigan et al., 2009). Synaptic plasticity plays a crucial role in perceiving, evaluating, and storing information, enabling adaptive responses to stimuli. Significantly, synaptic plasticity emerges as a key determinant in both short‐ and long‐term memory processes (Duman et al., 2016). In parallel, studies highlight shared neuroplasticity changes in chronic pain and depression, shedding light on their distinct characteristics (Ru et al., 2022; Sheng et al., 2017). Evidence from these studies elucidates that chronic pain can lead to neuroplastic alterations in neural circuits, contributing to depressive symptoms. However, the underlying mechanisms remain to be elucidated. BDNF, an intrinsic neurotrophic factor predominantly synthesized within the brain, exhibits prominent expression in the hippocampus and cerebral cortex (Du et al., 2020). Functionally, it assumes a crucial role in facilitating neurogenesis, neuronal differentiation, and synaptic plasticity through its interaction with its receptor tyrosine‐protein kinase B (TrkB) (Lu et al., 2014). Activation of the BDNF/TrkB signaling cascade subsequently triggers phosphorylation of the transcription factor cAMP response element binding protein (CREB) (Wang et al., 2018). Several studies have provided evidence on the therapeutic potential of targeting the BDNF/TrkB/CREB signaling pathway in ameliorating chronic pain and depressive symptoms (Price & Inyang, 2015; Ye et al., 2017).

The clinical management of comorbid chronic pain and depression typically involves combining analgesics and antidepressants. Nevertheless, the synergistic mechanism underlying this treatment approach remains elusive, and long‐term drug utilization raises concerns about dependency and adverse effects (Lottering & Lin, 2021; Obata, 2017). Therefore, viewing CIPD as a unified disease entity holds promise for developing safe and dependable therapeutic interventions. Electroacupuncture (EA) entails the application of electrical currents to acupuncture needles, augmenting its treatment efficacy when compared to conventional acupuncture. It is extensively employed worldwide to address pain and emotional disorders (Wu et al., 2019). Preclinical studies have demonstrated the efficacy of EA in alleviating pain symptoms and ameliorating depression‐like behavior in CIPD rats (Huang et al., 2020; Liao & Lin, 2021). In addition, EA may improve chronic neuropathic pain and depression‐like behaviors by modulating the CREB‐5‐hydroxytryptamine (5‐HT)/BDNF signaling pathway (Cong et al., 2021). What's more, acupuncture points LI4 and LR3 are frequently used in clinical and animal experiments to manage chronic pain or depression. These points work by activating neurons and targeting specific brain regions (Dias et al., 2016; Fan et al., 2016; Gao et al., 2023; Kasim & Viventius, 2022; Luo et al., 2017; Shan et al., 2014). However, the precise mechanism by which EA modulates the BDNF/TrKB/CREB signaling pathway to treat CIPD rats remains elusive. Based on the hypothesis that alterations in synaptic plasticity occur in rats with CIP, we postulated that EA could modulate hippocampal synaptic plasticity through the upregulation of the BDNF/TrKB/CREB signaling pathway, thereby ameliorating CIPD. Importantly, the findings from this study provide valuable experimental evidence to support the clinical utilization of EA as a therapeutic approach for patients suffering from CIPD.

## 2 MATERIALS AND METHODS

### 2.1 Animals

Some 8‐week‐old male Sprague–Dawley rats weighing 220–280 g were obtained from the Changsha Tianqin Biotechnology Co. [license No. SCXK (Xiang) 2019‐0014]. The rats were housed in plastic cages (five animals per cage) under a 12 h light/dark cycle with free access to water and food. The animal room was maintained at a constant temperature range of 23–25°C and a relative humidity range of 50%–60%. All rats were acclimated for 1 week before the start of the experiment to minimize the impact of environmental stress. All experiments were approved by the Guangxi University of Chinese Medicine Institutional Welfare and Ethical Committee (approval No. 20220523‐106). The 60 rats were randomly assigned to 4 groups: 0.9% normal saline (N.S.), complete Freund's adjuvant (CFA), CFA + duloxetine (Dulox), and CFA + EA groups (*n* = 15 per group).

### 2.2 CIPD model establishment

**FIGURE 1:**
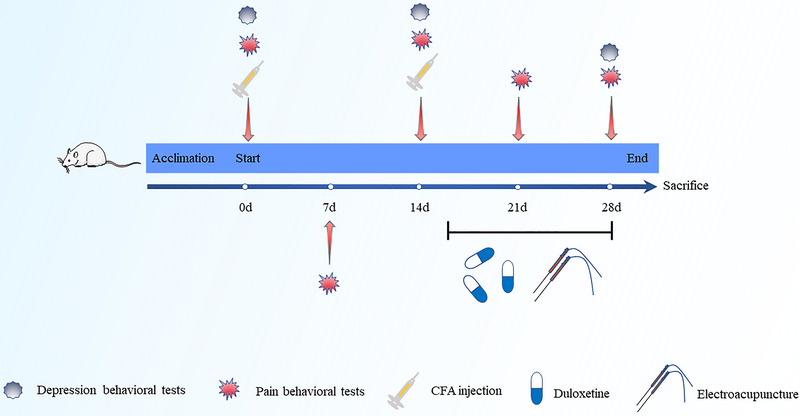
The process for the experiment.

The CIPD model was established according to Zhou et al. (2019) and Shao et al. (2021.) methods. Under 2% isoflurane (RWD Biotechnology Co., Ltd.) anesthesia, rats were positioned in a prone position and injected with 100 µL of CFA (Sigma) on the plantar surface of the left hind paw on day 0, followed by a 50µL injection on day 14, using a microinjector. The 0.9% N.S. group received an equivalent volume of 0.9% N.S. injection. The process of the experimental design is shown in Figure 1.

### 2.3 Electroacupuncture and duloxetine treatment

**FIGURE 2:**
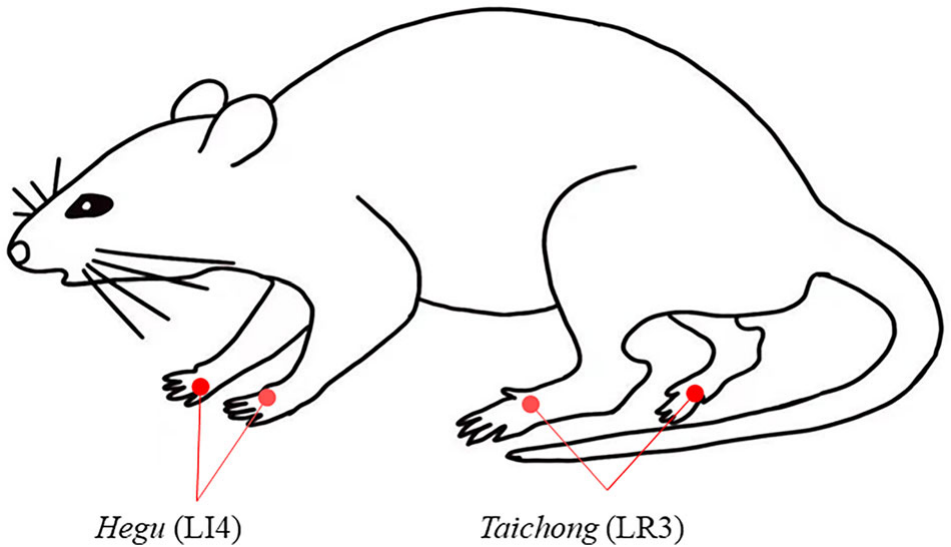
Schematic diagram of *Hegu* (LI4) and *Taichong* (LR3) acupoints.

From days 15 to 28, the model rats received EA and Dulox treatments. The acupoints *Hegu* (LI4) and *Taichong* (LR3) were bilaterally selected based on published literature (Kang et al., 2021). Using sterile disposable acupuncture needles (0.25 mm × 13 mm), the acupoints were inserted to a depth of 3–5 mm under inhalation anesthesia. A G6805‐I electronic pulse therapy instrument (Xinsheng Industrial Co., Ltd.) was then used to connect the acupoints on the same side, delivering a continuous wave with a frequency of 2/15 Hz and an intensity of 1 mA for 20 min per session. The location of the LI4 and LR3 is illustrated in Figure 2. For Dulox treatment, Dulox hydrochloride capsules (H20150287, Eli Lilly and Company) were dissolved in distilled water and administered orally at an intragastric dose of 10 mg kg.

### 2.4 Mechanical withdrawal threshold (MWT)

The mechanical withdrawal threshold (MWT) in rats was evaluated using the modified up–down method (Chaplan et al., 1994). Rats were placed in a polypropylene resin box with a metal screen floor and allowed to acclimate for 15 min before testing. A series of Von Frey filaments with increasing levels of force (0.4, 0.6, 1.0, 2.0, 4.0, 6.0, 8.0, 15.0, and 26.0) were applied vertically to the skin in the middle part of the left hind paw. The force of the filaments was gradually increased until they formed a “C” or “S” pattern, and a positive reaction was recorded if the rat displayed foot lifting, foot licking, or avoidance behavior. The MWT value was calculated using the formula: MWT = (10^[Xf + kδ]^)/10,000, where Xf represents the force value of the last filament used; *k* corresponds to the value on the lookup table that matches the positive responses observed; and *δ* represents the mean difference between stimuli used (0.224).

### 2.5 Thermal withdrawal latency (TWL)

Before nociceptive stimulation with a hot plate, rats were placed in a plexiglass box covered with a baffle for 15 min to allow for acclimation. The heat‐induced paw withdrawal latency was defined as the time from the beginning of stimulation to the appearance of leg raising and avoidance. A 10‐min interval was allowed between each stimulation to prevent sensitization to the hot plate. The same location was stimulated in each trial to ensure consistency of results. Latency time was recorded and averaged based on three consecutive measurements in each rat.

### 2.6 Open field test (OFT)

To assess the locomotion activity and exploratory behavior of the rats, a plain and black open field box (50 cm × 50 cm × 40 cm) was used in the study. After a 15‐min habituation period, each rat was placed in the central area of the box, and their motor behavior was recorded for 5 min using a video camera. The time spent in the central region and the total distance traveled by each rat were calculated based on the recorded videos using the SMART 3.0 software (Panlab).

### 2.7 Sucrose preference test (SPT)

Before the experiment, rats in each group were trained to adapt to sucrose water in a quiet room with low lighting. For the first 24 h, each rat was given two bottles of water containing 1% sucrose water with equal volumes to complete the training. For the second 24 h, one of the bottles containing sucrose water was replaced with an equal volume of distilled water, and each rat began fasting. On the next day, the consumption of both sucrose water and distilled water was measured. The sucrose water preference was calculated as follows: sucrose water preference (%) = sucrose water consumption (mL)/total liquid (sugar water + distilled water) consumption (mL) × 100%. The sugar water preference rate was calculated separately for each group of rats.

### 2.8 Forced swimming test (FST)

The forced swimming test (FST) is a widely utilized evaluation of depression‐like behaviors in rodents. Experimental rats were placed within an uncovered, transparent Plexiglas container with a water depth of approximately 30 cm and kept at a consistent temperature of 20 ± 2°C. Using a video camera, the cumulative immobility time of each rat was recorded over a 5‐min period, where immobility was defined as the absence of all limb movements except for some slight limb movements necessary for keeping the head above water and the nostrils free for breathing. Following the test, each rat was dried promptly and returned to the cage.

### 2.9 Nissl staining (NS)

Following the dehydration of the tissues with gradient alcohol solutions (75%, 85%, 90%, and 95%), they were sectioned using a frozen sectioning machine. The sections were then incubated with pre‐warmed toluidine blue Nissl staining solution for 20–40 min, subsequently decolorized with an alcohol gradient (2 min per step), and immediately washed with distilled water for 30 s. Finally, the sections were sealed with neutral resin, and the pathological morphology of hippocampal neurons using light microscopy.

### 2.10 Transmission electron microscopy (TEM) analysis

The hippocampal tissues were initially fixed with an electron microscope fixing buffer and stored at 4°C. Following washing with 0.1 M phosphate‐buffered saline (PBS), the tissues were further fixed using 1% O_s_O_4_ for 2 h at room temperature. Subsequent dehydration was facilitated using a gradient approach of ethanol and acetone, and the subject tissues were ultimately embedded within SPI‐Pon812 resin. Ultrathin sectioning (50–70 nm) was performed utilizing an ultramicrotome, with the resulting tissue slices double‐stained with both uranyl acetate and lead citrate. Ultrastructural alterations of hippocampal neuronal synapses were then visualized and investigated using electron microscopy (HT7700, Hitachi). Subsequently, synaptic structures were evaluated using ImageJ software.

### 2.11 Enzyme‐linked immunosorbent assay (ELISA)

Hippocampal levels of serotonin (5‐HT) and γ‐aminobutyric acid (GABA) were measured through the use of the universal 5‐HT enzyme‐linked immunosorbent assay (ELISA) kit (E‐EL‐0033, Elabscience) and the rat GABA ELISA kit (H168, Nanjing Jiancheng). Tissues were first washed with pre‐chilled PBS, weighed, and clipped to yield 50 mg of hippocampal tissue. The tissue was then homogenized using a glass homogenizer in 450 µL of PBS and centrifuged at 5000 × *g* for 10 min at 4°C. Sample diluent (50 µL) was added to each standard well and sample blank well, followed by the addition of 50 µL of the corresponding tissue sample. Next, 100 µL of HRP‐conjugated reagent was added to each well and incubated for 45 min at 37°C, after which 100 µL of enzyme conjugate working solution was added per well and then incubated at 37°C for 30 min. Finally, 50 µL of stop solution was added to each well, and the OD values at 450 nm were measured by an enzyme standardization instrument. The concentrations of GABA and 5‐HT within the hippocampal tissues were calculated according to the corresponding standard curves.

### 2.12 Ultraviolet colorimetry method (UCM)

The measurement of total protein content enables a more precise calculation and expression of glutamate acid (Glu) transmitter concentrations. To assess levels of Glu, the Glu Assay Kit and total protein assay kit (A074‐1‐1, A045‐2‐1, Nanjing Jiancheng), respectively, were utilized according to the manufacturer's instructions. Briefly, 50 mg of rat hippocampus tissue was weighed and added to 450 µL of 0.9% N.S. for mechanical homogenization within an ice bath. The homogenate was then centrifuged at 2500 rpm for 10 min, and the total protein level was analyzed. In addition, 0.2 mL of the supernatant was collected while adding 0.6 mL of reagent I. The mixture was then subjected to a further centrifugation step at 3000–3500 rpm for 10 min, and 0.5 mL of the supernatant was analyzed for glutamate. Glu concentration was determined using a ultraviolet–VIS spectrophotometer (GENESYS5, Milton Roy Company).

### 2.13 Western blot (WB) analysis

Some 50mg hippocampal tissues were taken, ground, and added to RIPA lysate (R0020, Solarbio Co., Ltd.) before undergoing homogenization and subsequent centrifugation at 4°C. The collected supernatant was then subjected to protein content determination via the BCA method. Sample proteins of 20 µg were resolved by 10% SDS–PAGE and transferred to polyvinylidene difluoride membranes (IPVH00010, Millipore), which were then sealed with 5% nonfat milk in Tris‐buffered saline containing 0.1% Tween‐20 (TBST) for 1 h at room temperature. Primary antibodies anti‐postsynaptic density protein‐95 (PSD‐95) (1:1500, DF12274, Affinity), anti‐synuclein (Syn) (1:1000, AF0402, Affinity), anti‐BDNF (1:1200, AB108319, Abcam), anti‐TrKB (1:1000, AF6461, Affinity), anti‐CREB (1:1000, AF3189, Affinity), and β‐actin (1:6000, AF7018, Affinity) were then incubated overnight at 4°C. The membranes were then washed for 10 min each, three times over, with TBST before further incubation with the horseradish peroxidase–conjugated anti‐rabbit secondary antibody (ZB2301, ZSGB‐BIO) for 90 min at room temperature. Following further washes with TBST, immunoreactive bands were detected using enhanced chemiluminescence, and protein band analysis was undertaken to utilize ImageJ software (National Institutes of Health) to obtain grayscale values for the relative protein content of the target protein bands/β‐actin protein bands.

### 2.14 Quantitative real‐time PCR (qRT‐PCR) assay

Total RNA was extracted from 100 mg of hippocampal tissue per rat using the Trizol method. Briefly, the tissue was ground in liquid nitrogen and 1 mL of Trizol was added, followed by homogenization and 5 min of settling. Next, 0.2 mL of chloroform was added, and the mixture was centrifuged at 4°C for 15 min at 12,000 × *g*. The supernatant (0.5 mL) was then transferred to a clean 1.5 mL centrifuge tube, mixed with 0.5 mL of isopropanol, and centrifuged again at 4°C for 10 min at 12,000 × *g*. The supernatant was discarded, and the precipitate was washed with 1 mL of 75% ethanol before being dried in a metal bath at 65°C. The RNA was dissolved by adding DEPC water, and the concentration was measured using an ultra‐micro nucleic acid detector. Total RNA was reverse‐transcribed to cDNA according to the instructions of the reverse transcription kit. The primer sequences for qRT‐polymerase chain reaction (PCR) are listed in Table 1, with a reaction procedure of pre‐denaturation at 95°C for 30 s, followed by 40 cycles of 95°C for 10 s and 60°C for 30 s. After completion, the comparative threshold (Ct) cycle of each reaction well was detected by PCR instrument, and the relative expression of BDNF and TrkB mRNA was calculated using the 2^−ΔΔ^ *^Ct^* method with the β‐actin gene as the internal reference control.

**TABLE 1:** Primer sequences.

Gene name	Primer sequence (5′ to 3′)	Product length (bp)
BDNF	Upstream: ACGGTCACAGTCCTGGAGAAAG	154
	Downstream: ACGATTGGGTAGTTCGGCATT	
TrKB	Upstream: GGGCTTATGCTTGCTGGTCTT	150
	Downstream: TCTGGGTCAATGCTGTTAGGTT	
β‐actin	Upstream: AGATTACTGCCCTGGCTCCTAG	144
	Downstream: CATCGTACTCCTGCTTGCTGAT	

### 2.15 Immunofluorescence (IF) staining

The hippocampal tissue was fixed in 4% paraformaldehyde, followed by routine paraffin embedding. Subsequent sections were sliced to a thickness of 5 µm and were dewaxed and underwent high‐pressure citric acid antigen retrieval. After being washed with distilled water for 5 min, the sections were soaked in 0.1 M PBS for 1 min and then blocked in 5% BSA for 30 min at room temperature. Next, the tissue blocks were subjected to blocking with 5% BSA for 30 min at room temperature. Primary antibodies anti‐PSD‐95 (1:150, DF12274, Affinity) and anti‐Syn (1:100, AF0402, Affinity) were then added dropwise and incubated overnight at 4°C. After washing three times with PBS for 5 min each, secondary antibodies (ZF0511, ZF0516, ZSGB‐BIO) were similarly added and incubated for 30 min at 37°C. Following 15 min of PBS rinsing, DAPI was added dropwise and incubated for 10 min at room temperature. A confocal laser scanning fluorescence microscope was used to detect and analyze all stained sections.

### 2.16 Statistical analysis

Experimental data analysis was conducted using SPSS 26.0 statistical software (IBM Corp.). All results were expressed as a mean ± standard error of the mean. The normality of data distribution was assessed using the Shapiro–Wilk normality test. For the analysis of multiple time points, we employed repeated measures analysis of variance (ANOVA), whereas one‐way ANOVA with Tukey's post hoc test was used for the analysis of multiple groups. In instances where variance was unequal, we used Dunnett's *T*3 test. The *p* value <.05 was considered statistically significant.

## 3 RESULTS

### 3.1 EA alleviates CFA‐induced mechanical and thermal hyperalgesia behavioral responses in rats

**FIGURE 3:**
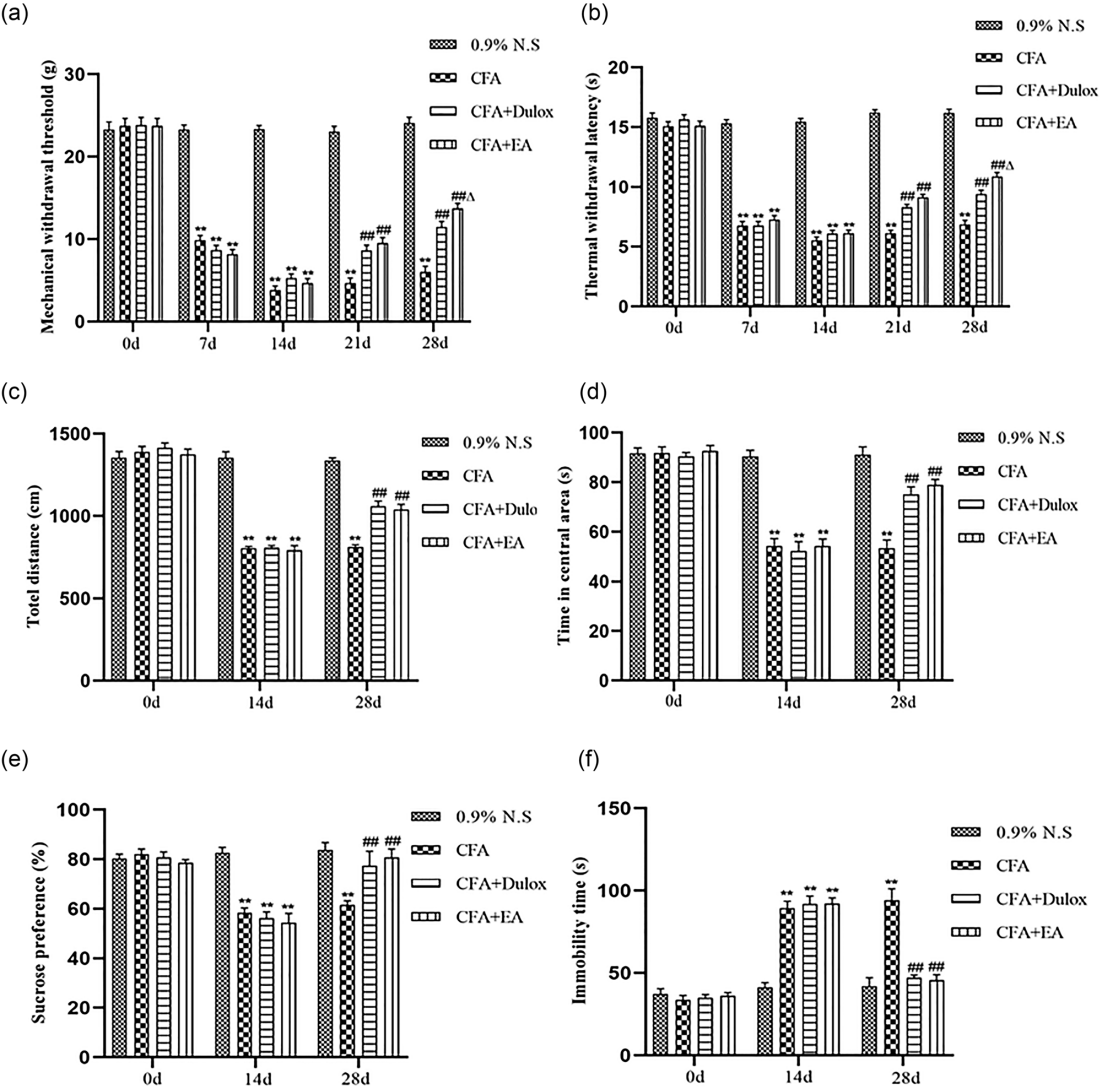
Electroacupuncture (EA) improves hyperalgesia and depressive behaviors in chronic inflammatory pain depression (CIPD) rats. (a) Changes in mechanical withdrawal threshold (MWT) of rats in each group at multiple time points; (b) changes in thermal paw withdrawal latency (TWL) of rats in each group at multiple time points. Data are expressed as mean ± standard error of the mean (SEM) (*n* = 15). ^**^ *p* < .01 versus the 0.9% normal saline (N.S.) group; ^##^ *p* < .01 versus the complete Freund's adjuvant (CFA) group; ^Δ^ *p* < .05 versus the CFA + duloxetine (Dulox) group. (c) Total distance traveled by rats in the open field; (d) the time spent in the central area of rats in the open field; (e) sucrose preference of rats in each group; (f) duration of immobility spent in the forced swimming test (FST). Data are expressed as mean ± SEM (*n* = 5). ^**^ *p* < .01 versus the 0.9% N.S. group; ^##^ *p* < .01 versus the CFA group.

CFA injection in the plantar region produces a rapid reduction in both mechanical and thermal hyperalgesia, indicating the onset of nociceptive sensitization. In this study, EA elicits a significant improvement in mechanical and thermal hyperalgesia in rats with CIPD. As shown in Figure 3a,b, baseline levels of MWT and thermal paw withdrawal latency (TWL) were similar across all groups before CFA injection (*p* > .05). Compared with the 0.9% N.S. group, both the MWT and TWL significantly decreased at days 7 and 14 after induction (*p* < .01). From days 15 to 28, EA stimulation resulted in a significant increase in both the MWT and TWL in CIPD rats (*p* < .01). Notably, on day 28, the CFA + EA group demonstrated significantly greater improvement in both MWT and TWL compared with the CFA + Dulox group (*p* < .05). This finding suggests that EA may have a superior long‐term analgesic effect in CIPD rats when compared to Dulox.

### 3.2 EA alleviates CIP‐induced depression‐like behaviors in rats

Chronic and persistent inflammatory pain is known to induce depression‐like behaviors. To assess the extent of these behaviors, we conducted three behavioral experiments: the open field test (OFT), sucrose preference test (SPT), and FST. As seen in Figure 3c–f, all groups showed similar baseline levels in the OFT, SPT, and FST (*p* > .05). By day 14, compared with the 0.9% N.S. group, the CFA group exhibited significant depressive behaviors, which confirmed the successful establishment of the CIPD model. By day 28, the CFA group showed a significant increase in total distance traveled and time stayed in the central area, a decreased sucrose consumption in the SPT, and an increase in immobility time during the FST compared with the 0.9% N.S. group (*p* < .01). In comparison with the CFA group, the CFA + Dulox and CFA + EA groups exhibited decreased immobility time in FST, alongside a substantial increase in the total distance traveled, time stay in the central area, and sucrose intake of CIPD rats (*p* < .01).

### 3.3 EA ameliorates morphological abnormalities and preserves synaptic ultrastructure of neural cells and in the hippocampus of rats with CIPD

**FIGURE 4:**
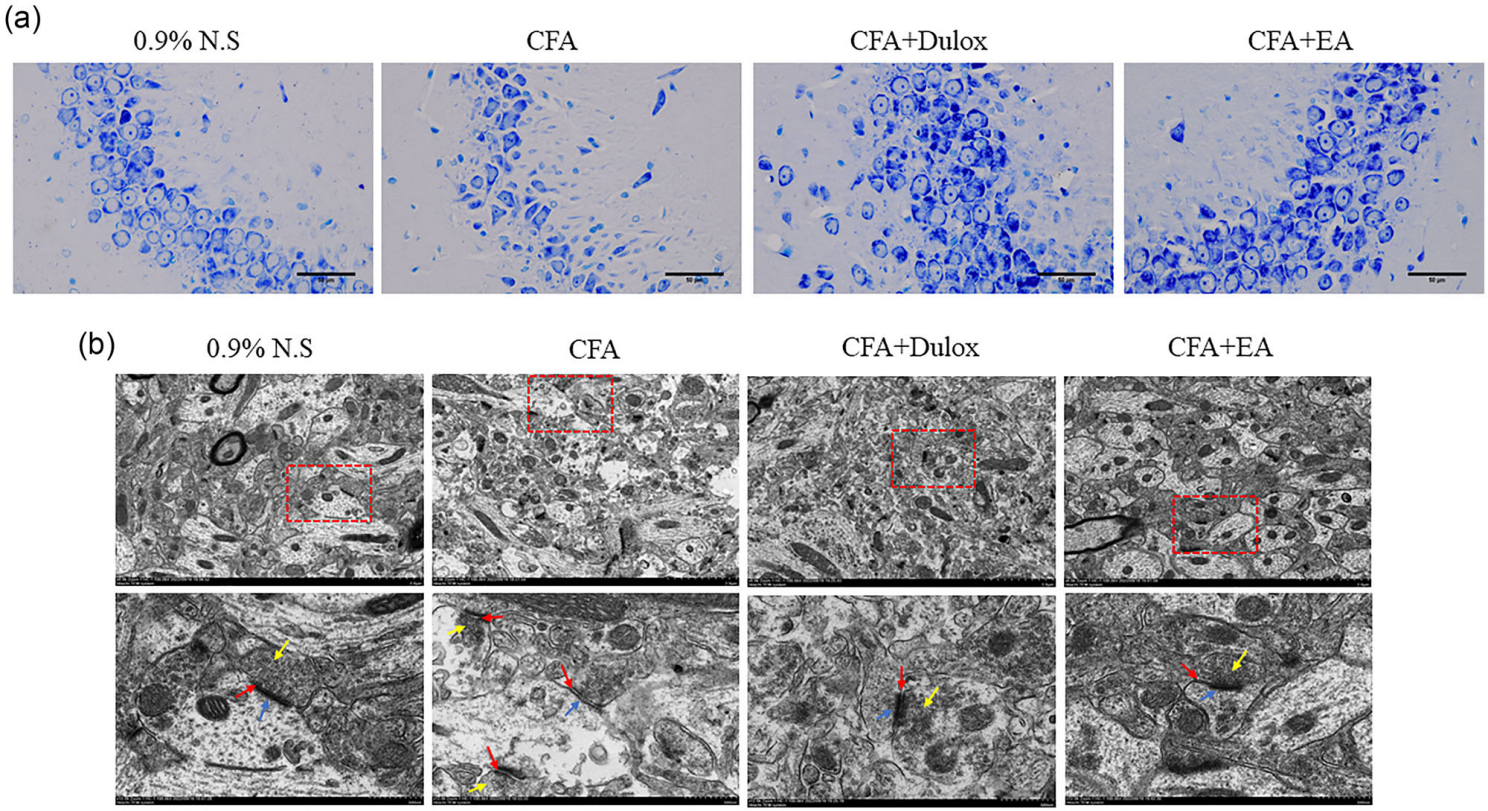
Electroacupuncture (EA) ameliorated hippocampal morphological and synaptic ultrastructure changes under chronic inflammatory pain depression (CIPD). (a) Representative images of the hippocampal Nissl staining of each group. Scale bar: 50 µm. (b) Representative images of the hippocampal synaptic ultrastructure of each group. Synaptic cleft (red arrow); thickness of postsynaptic density (blue arrow); synaptic vesicle (yellow arrow); scale bar: 1 or 2 µm.

We conducted a comparative analysis of the hippocampal neurons in the 0.9% N.S. group and the CFA group to determine differences in neuronal morphology. The 0.9% N.S. group showed no signs of abnormal morphology with a clear structure and orderly arrangement of neurons. In contrast, the CFA group displayed sparse and damaged neurons leading to considerable cell loss. However, in both the CFA + Dulox and CFA + EA groups, hippocampal neurons exhibited a reduction in cell loss, characterized by a neater arrangement of cells and clearer nuclei (Figure 4a). This evidence supports a neuroprotective effect of both CFA + Dulox and CFA + EA groups on hippocampal neurons in CIPD.

To assess the impact of EA on the morphological structure of the hippocampus, we employed transmission electron microscopy to observe the synaptic ultrastructure. In the 0.9% N.S. group, synaptic vesicles were evenly distributed, and no significant changes were observed in the postsynaptic density or synaptic cleft. In contrast, the CFA group exhibited a decrease in the number of synaptic vesicles, shrinkage in the postsynaptic density, and a significant increase in the synaptic cleft, indicative of inhibited neural signaling. However, both the CFA + Dulox and CFA + EA groups demonstrated an increase in the number of synaptic vesicles, a thickening of the postsynaptic density, and a reduction in the synaptic cleft when compared to the CFA group (Figure 4b).

### 3.4 EA regulates the content of neurotransmitters in the hippocampus of rats with CIPD

**FIGURE 5:**
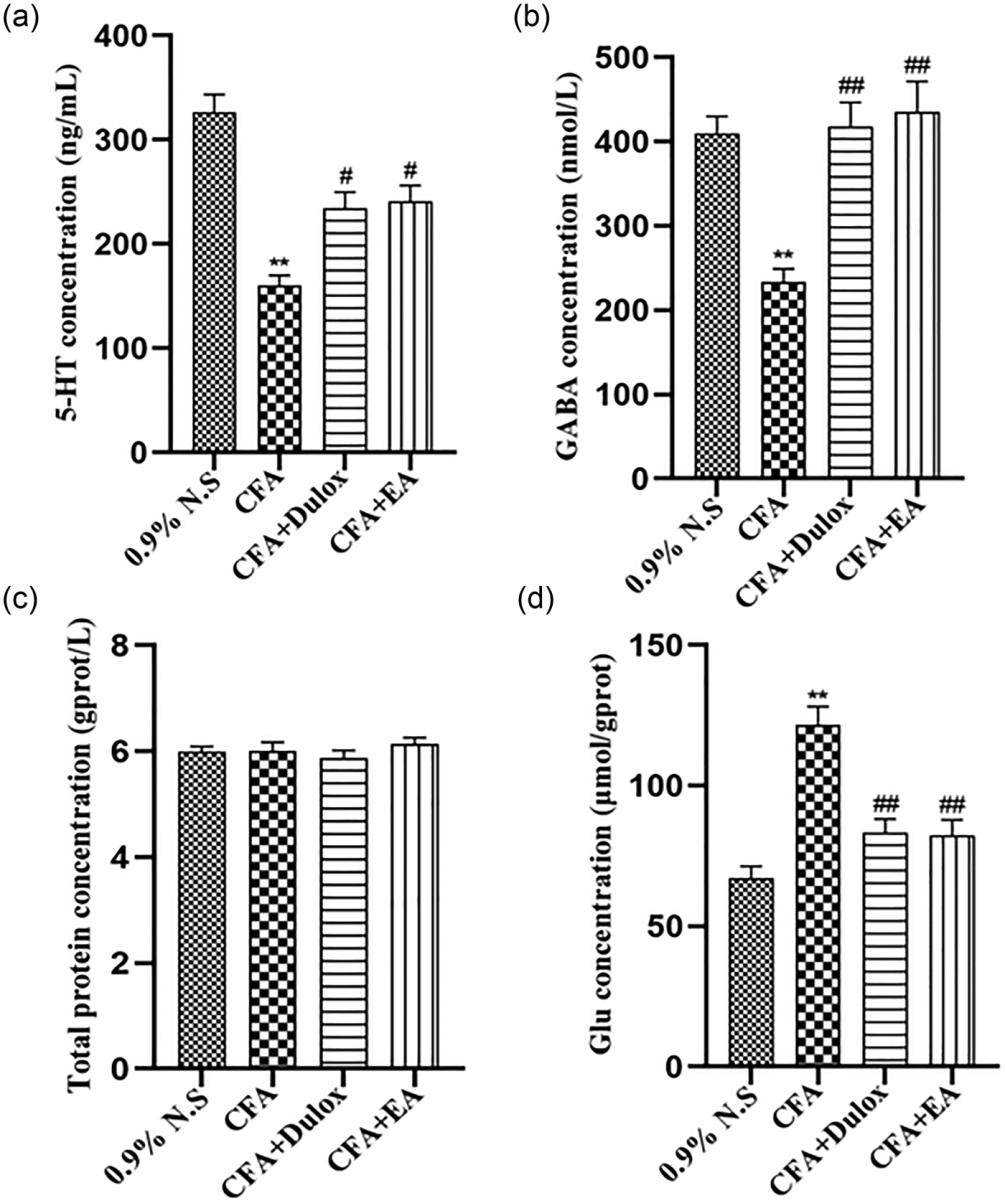
Electroacupuncture (EA) regulates the expression of hippocampal neurotransmitters under chronic inflammatory pain depression (CIPD). (a) The concentration of 5‐hydroxytryptamine (5‐HT); (b) the concentration of γ‐aminobutyric acid (GABA); (c) the concentration of total protein; (d) the concentration of glutamic (Glu). Data are expressed as mean ± standard error of the mean (SEM) (*n* = 5). ^**^ *p* < .01 versus the 0.9% normal saline (N.S.) group; ^#^ *p* < .05, ^##^ *p* < .01 versus the complete Freund's adjuvant (CFA) group.

Neurotransmitters play an important role in long‐term hippocampal synaptic plasticity, which is considered to be involved in chronic pain and depression (Palacios‐Filardo & Mellor, 2019; Sheng et al., 2017). As shown in Figure 5, compared with the 0.9% N.S. group, the CFA group demonstrated a decrease in hippocampal levels of 5‐HT and GABA (*p* < .01), accompanied by an increase in Glu levels (*p* < .01). EA and Dulox treatment significantly increased the production of 5‐HT and GABA (*p* < .05, *p* < .01) while decreasing the production of Glu in the hippocampus (*p* < .01).

### 3.5 EA upregulates the expression of synapse‐associated proteins in the hippocampus of rats with CIPD

**FIGURE 6:**
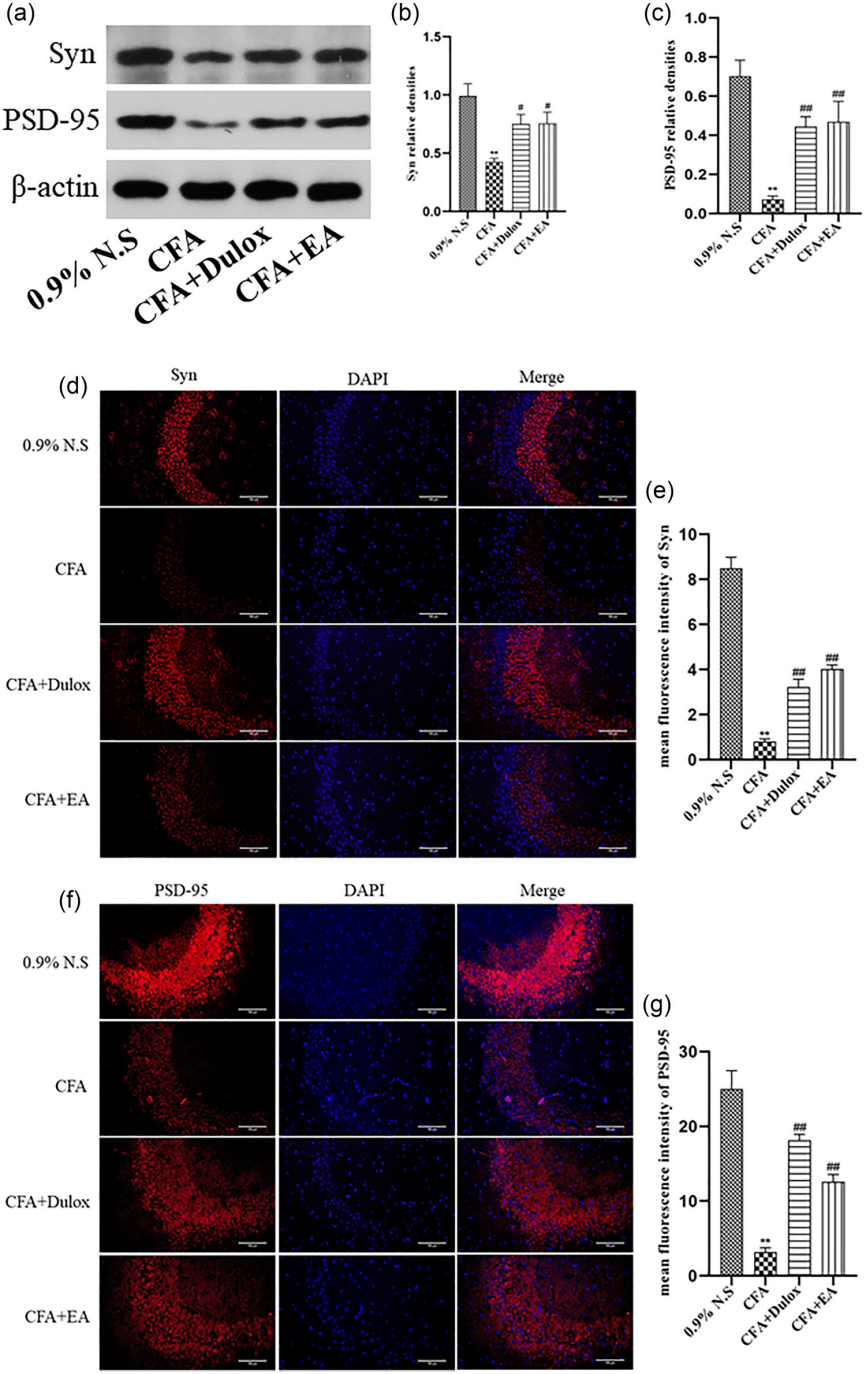
Electroacupuncture (EA) increases the expression of synapse‐associated proteins in the hippocampus under chronic inflammatory pain depression (CIPD). (a) Western blot analysis of synuclein (Syn), postsynaptic density protein‐95 (PSD‐95) levels in the hippocampus, with β‐actin as the loading control. (b) Quantification of Syn protein expression level. (c) Quantification of PSD‐95 protein expression level. (d) Colocalization of Syn in the hippocampus. Scale bar: 50 µm. (e) Mean fluorescence intensity of Syn. (f) Colocalization of PSD‐95 in the hippocampus. Scale bar: 50 µm. (g) Mean fluorescence intensity of PSD‐95. Data are expressed as mean ± standard error of the mean (SEM) (*n* = 3). ^**^ *p* < .01 versus the 0.9% normal saline (N.S.) group; ^##^ *p* < .01 versus the complete Freund's adjuvant (CFA) group.

Synapse‐associated proteins, including Syn and PSD‐95, play crucial roles in plasticity and inflammatory pain (Yamada et al., 2012; Zhang et al., 2019). In this study, we utilized western blotting (WB) analysis to assess the expression levels of hippocampal synapse–associated proteins. As depicted in Figure 6a–c, the WB analysis revealed a significant reduction in the levels of Syn and PSD‐95 in the hippocampal tissues compared to the 0.9% N.S. group (*p* < .01). However, treatment with EA and Dulox led to a notable increase in the levels of Syn (*p* < .05) and PSD‐95 (*p* < .01). Furthermore, we utilized immunofluorescence (IF) staining to examine the expression of synapse‐associated proteins in the hippocampus. Similarly, as shown in Figure 6d–g, IF staining demonstrated a significant decrease in the levels of Syn and PSD‐95 in the CFA group compared to the 0.9% N.S. group (*p* < .01). In contrast, both the CFA + Dulox and CFA + EA groups exhibited a significant increase in the expression levels of synapse‐associated proteins compared to the CFA group (*p* < .01).

### 3.6 EA activates the BDNF/TrKB/CREB signaling pathways in the hippocampus of rats with CIPD

**FIGURE 7:**
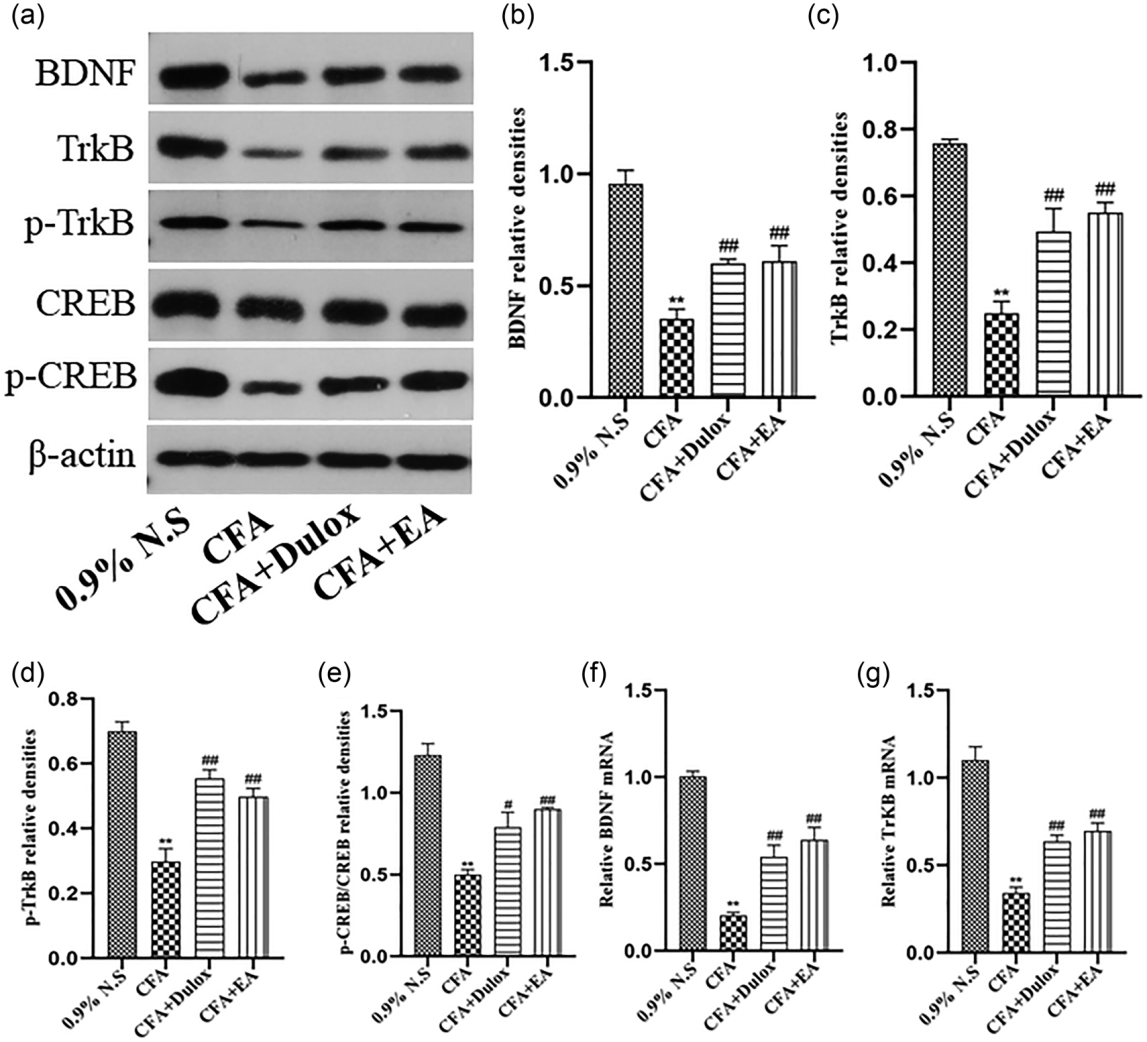
Electroacupuncture (EA) upregulates brain‐derived neurotrophic factor (BDNF)/tyrosine–protein kinase B (TrKB)/cAMP response element binding protein (CREB) protein and gene expression in the hippocampus under chronic inflammatory pain depression (CIPD). (a) Western blot analysis of BDNF, TrKB, p‐TrKB, CREB, and p‐CREB levels in the hippocampus, with β‐actin as the loading control. (b) Quantification of BDNF protein expression level. (c) Quantification of TrKB protein expression level. (d) Quantification of p‐TrKB protein expression level. (e) Quantification of p‐CREB/CREB relative expression level. (f) Relative expression level of BDNF mRNA in the hippocampus. (g) Relative expression level of TrKB mRNA in the hippocampus. Data are expressed as mean ± standard error of the mean (SEM) (*n* = 3). ^**^ *p* < .01 versus the 0.9% normal saline (N.S.) group; ^##^ *p* < .01 versus the complete Freund's adjuvant (CFA) group.

Recent studies have established a connection between synaptic plasticity and the BDNF/TrKB/CREB signaling pathway (Wang et al., 2023; Zhao et al., 2021). Furthermore, increasing its expression has shown promise in ameliorating symptoms of depression and chronic pain (Cong et al., 2021). In this study, the WB analysis results indicated a significant reduction in the expression levels of BDNF, TrKB, p‐TrKB, and p‐CREB/CREB proteins in the hippocampus of the CFA group compared to the 0.9% N.S. group (*p* < .01). However, treatment with Dulox and EA effectively reversed this inhibitory effect, restoring the expression levels of BDNF, TrKB, p‐TrKB, and p‐CREB/CREB proteins (*p* < .01, *p* < .05) (Figure 7a–e). Besides, the qRT‐PCR assay revealed a significant decrease in the expression levels of BDNF mRNA and TrKB mRNA in the hippocampus of the CFA group compared to the 0.9% N.S. group (*p* < .01). Notably, both Dulox and EA treatments resulted in a substantial increase in the expression levels of BDNF mRNA and TrKB mRNA compared to the CFA group (*p* < .01). These findings indicate that EA has the potential to upregulate the expression of the BNDF/TrKB/CREB signaling pathway in the hippocampus of CIPD rats.

## 4 DISCUSSION

In this study, the CIPD rat model was established by administering two plantar injections of CFA. CFA is a water‐in‐oil emulsion containing inactivated *Mycobacterium tuberculosis*, which serves to enhance the immunogenicity of the antigen (All et al., 2010). CFA plantar injection is a well‐established method for inducing an inflammatory pain model in rats that closely mirrors the characteristics of CIP observed in humans. The CFA model elicits sustained mechanical and thermal pain hyperalgesia in rats, with effects lasting beyond a duration of 3 weeks (Kremer et al., 2021). This model is extensively utilized to investigate the comorbidity between inflammatory pain and depression disorders. A study has reported that pain caused by CFA injections can induce depression‐like behaviors at week 4 (Huang et al., 2020). Zhang et al. (2016) demonstrated that the manifestation of depression‐like behavior could arise as early as 7 days subsequent to the intra‐articular administration of CFA in the tibiotarsal joint cavity. These studies demonstrate that the injection of CFA rapidly induces pain and depressive behaviors in rodents. Additionally, the dosage of CFA also influences the time needed for modeling.

EA has been proven effective in alleviating CIPD in clinical settings. In recent years, some preclinical studies have elaborated the mechanism of EA on CIPD. Huang et al. (2020) discovered that EA effectively alleviates both pain and depression in the CIPD model by the upregulation of the *N*‐methyl‐d‐aspartate (NMDA) receptor signaling pathway in the prefrontal cortex (mPFC), hippocampus, and hypothalamus. Further investigations have revealed that EA effectively mitigates CIPD by enhancing the expression of transient receptor potential V1 (TRPV1) in the mPFC, hypothalamus, and periaqueductal gray. This has also been observed in TRPV1 knockout models, indicating that TRPV1 serves as a shared target involved in the development of CIP as well as depression (Liao & Lin, 2021). Our previous study has additionally validated the efficacy of EA in diminishing hippocampal apoptosis through the activation of the phosphatidylinositol 3‐kinase/Artesunate (PI3K/Akt) signaling pathway (Yang et al., 2023). It is noted that CIP caused by CFA injection not only leads to depression but also induces anxiety symptoms. EA effectively mitigates both CIP and anxiety by activating parvalbumin neurons in the anterior cingulate cortex (Shao et al., 2021), inducing an increase in neurotensin/neurotensin receptor expression (Du et al., 2020), and inhibiting protein kinase Mzeta activity (Du et al., 2017). These findings provide insight into the mechanisms by which EA intervenes in CIP and negative emotion comorbidity, based on which we found that EA activates the BDNF/TrKB/CREB pathway to regulate synaptic plasticity of the hippocampus and alleviate pain and depression‐like behaviors.

The hippocampus is an important part of the limbic system, which assumes a critical role in the regulation of emotion, memory, and cognition. Moreover, its significance as a pivotal site for the integration of pain and depression further underscores its complex involvement in understanding the interplay among these conditions. In rodent models of chronic pain, notable alterations are observed in the hippocampus, encompassing both morphological modifications and intricate changes in the expression of microscopic genes (Vasic & Schmidt, 2017). Correspondingly, analogous alterations manifest in rodent models of depression, giving rise to disturbances in synaptic morphology within distinct hippocampal subregions (Liu et al., 2017). Consistent with those findings, rats in the CFA group exhibited notable neuronal sparsity and damage, accompanied by cellular loss. Meanwhile, a decrease in the synaptic vesicle count, shrinkage of the postsynaptic density, and a significant increase in the synaptic cleft were observed. These findings indicate that CFA‐induced CIPD rats can potentially elicit morphological and structural alterations within the hippocampus.

The significant involvement of neurotransmitters in the etiology of chronic pain and depression has been widely recognized. Extensive investigation into the hypothesis of neurotransmitter dysregulation in the context of chronic pain and depression has been conducted for many years, yielding robust evidence that establishes a strong association between alterations in neurotransmitter systems and the co‐occurrence of chronic pain and depression (Sheng et al., 2017). It is widely recognized that alterations in the 5‐HT pathway, coupled with diminished 5‐HT levels in the hippocampal region, are prevalent manifestations in both chronic pain and depression (Mihailescu‐Marin et al., 2020; Zhang et al., 2016). Emerging evidence highlights the pivotal involvement of 5‐HT in the processing of pain signals, as well as its role within the cortical area responsible for emotion regulation (Haleem, 2019). Glu, a crucial excitatory neurotransmitter in the central nervous system, exhibits elevated release in response to noxious stimuli, subsequently activating postsynaptic ionotropic glutamate receptors, including NMDA and α‐amino‐3‐hydroxy‐5‐methyl‐4‐isoxazolepropionic acid (AMPA) (Sheng et al., 2017). Humo et al. (2019) have provided compelling evidence showcasing the efficacy of NMDA receptor antagonists and AMPA receptor promoters in effectively mitigating both pain and depressive symptoms. In addition, GABA, the principal inhibitory neurotransmitter in the nervous system, plays a pivotal role in mediating pain‐depression comorbidity. Intraperitoneal administration of alkylated analogs of GABA has demonstrated notable alleviation of injurious responses and deficits in pleasure sensing in partial sciatic nerve ligation mice (La Porta et al., 2016), thereby implying the involvement of the GABAergic system in pain‐depression comorbidity. These findings reveal that dysregulation of inter‐synaptic neurotransmitters contributes to the onset of both chronic pain and depression. In this study, we found that the content of 5‐HT and GABA in the hippocampus decreased significantly in CFA rats; on the contrary, Glu was increased.

Neurotransmitters play a vital role as catalysts in initiating cellular activation by engaging both G‐protein‐coupled receptors and growth factors that bind to TrkB receptors. This intricate process of binding subsequently culminates in the augmentation of CREB protein phosphorylation, a key mediator in triggering BDNF transcription within the hippocampus. Such transcription is paramount for the maintenance and regulation of synaptic function and activity (Duman & Voleti, 2012). These phosphorylation events are mediated through diverse pathways, including Ca^2+^‐dependent mechanisms, the PI3K/Akt pathway, and the mitogen‐activated protein kinase cascade (Malhi & Mann, 2018). In our present study, we found that the BDNF/TrKB/CREB pathway may mediate hippocampal synaptic plasticity, and activation of this pathway may be a therapeutic strategy for CIPD.

Synapse‐associated proteins, such as PSD‐95 and Syn, assume vital functions in synaptic plasticity as well as pain and depression (Shen et al., 2020; Zhang et al., 2019). In the context of chronic pain and depression, the crucial postsynaptic marker PSD‐95 interacts with the postsynaptic glutamate receptors, NMDA and AMPA (Gu & Zhu, 2020). Synaptic protein levels, particularly Syn, have been widely employed as reliable indicators of synaptic count and transmission efficacy. Recent studies have demonstrated that alterations in synaptic‐associated proteins, specifically PSD‐95 and Syn, play a pivotal role in modulating synaptic plasticity in response to pain and depression (Shen et al., 2020; Zhang et al., 2019), thus highlighting the intricate relationship between these molecular changes and the modulation of synaptic plasticity. Our study confirmed that EA upregulated the expression of synapse‐associated proteins.

Dulox, a selective serotonin‐norepinephrine reuptake inhibitor, has been clinically utilized for the management of chronic pain and depression (Raskin et al., 2007). Hence, Dulox was selected as the positive control drug in this study. Additionally, Dulox demonstrated the ability to ameliorate pain‐ and depression‐related behaviors in rats manifesting CIPD. Interestingly, our findings revealed that following 14 days of EA, rats in the CFA + EA group exhibited superior efficacy in terms of MWT and TWL when compared to the CFA + Dulox group. This finding suggests that EA may confer superior analgesic effects during prolonged treatment regimens.

This study establishes an experimental foundation for the clinical application of EA in managing CIPD. Additionally, it provides a rational basis for targeting acupoints LI4 and LR3 to alleviate CIPD symptoms. These findings support the use of EA as an adjunctive therapeutic option for patients and offer a valuable framework for its clinical implementation. However, there are some limitations to our present study. First, this study primarily examined the impact of the hippocampal region on CIPD rats. However, it is crucial to conduct additional research to elucidate the role of other brain regions in this complex relationship. Consequently, further research is warranted to unravel the contributions of these additional regions. Second, although the present study significantly contributes to our understanding of this phenomenon, it is imperative to highlight that pathway inhibitors were not employed for reverse validation, so we were unable to definitively establish the precise contribution of this signaling pathway in both the progression and management of the disease, leading to a degree of uncertainty in the obtained results. In future studies, it is recommended to augment the size of the pathway inhibitor group to provide further substantiation for the underlying mechanisms involved. Therefore, by addressing these limitations, we can enhance the robustness and reliability of our study.

## 5 CONCLUSION

In conclusion, our findings underscore the efficacy of both EA in alleviating pain and mitigating depression‐like symptoms in CIPD rats. These therapeutic effects are likely mediated through the activation of the BDNF/TrKB/CREB signaling pathway, thereby exerting regulatory influence on synaptic plasticity in the hippocampus.

## AUTHOR CONTRIBUTIONS

**Pu Yang**: Formal analysis; methodology and writing—original draft. **Haiyan Chen**: Data curation and investigation. **Tian Wang**: Data curation and investigation. **Hong Su**: Data curation and investigation. **Jing Li**: Data curation and investigation. **Yujun He**: Data curation and investigation. **ShengYong Su**: Conceptualization; supervision; writing—review and editing.

## CONFLICT OF INTEREST STATEMENT

The authors declare that they have no conflicts of interest.

### PEER REVIEW

The peer review history for this article is available at https://publons.com/publon/10.1002/brb3.3310.

## Data Availability

The data used to support the findings of this study are available from the published literature; further inquiries can be directed to the corresponding author.
